# Development and psychometric properties the Barriers to Access to Care Evaluation scale (BACE) related to people with mental ill health

**DOI:** 10.1186/1471-244X-12-36

**Published:** 2012-06-20

**Authors:** Sarah Clement, Elaine Brohan, Debra Jeffery, Claire Henderson, Stephani L Hatch, Graham Thornicroft

**Affiliations:** 1 Health Service and Population Research Department, Institute of Psychiatry, King’s College London, De Crespigny Park, London, SE5 8AF, UK; 2 Department of Psychological Medicine, Institute of Psychiatry, King's College London, Weston Education Centre, Cutcombe Road, London, SE5 9RJ, UK

**Keywords:** Measure, Barriers to care, Access, Health care seeking, Stigma, Psychometric

## Abstract

**Background:**

Many people with mental illness do not seek or delay seeking care. This study aimed to develop, and provide an initial validation of, a comprehensive measure for assessing barriers to access to mental health care including a ‘treatment stigma’ subscale, and to present preliminary evidence about the prevalence of barriers experienced by adults currently or recently using secondary mental health services in the UK.

**Methods:**

The Barriers to Access to Care Evaluation scale (BACE) was developed from items in existing scales, systematic item reduction, and feedback from an expert group. It was completed in an online survey by 117 individuals aged 18 and over who had received care from secondary mental health services in the past 12 months. Internal consistency, test-retest reliability, convergent validity (correlation of treatment stigma subscale with the Stigma Scale for Receiving Psychological Help (SSRPH) and with the Internalised Stigma of Mental Illness Scale (ISMI)), respondent opinion and readability were assessed.

**Results:**

The BACE items were found to have acceptable test-retest reliability as all but one of the items exceeded the criterion for moderate agreement. The treatment stigma subscale had acceptable test-retest-reliability and good internal consistency. As hypothesised the subscale was significantly positively correlated with the SSRPH and the ISMI demonstrating convergent validity. The developmental process ensured content validity. Respondents gave the BACE a median rating of 8 on the 10-point quality scale. Readability scores indicated the measure can be understood by the average 11 to 12 year-old. The most highly endorsed barrier was ‘concern that it might harm my chances when applying for jobs’. The scale was finalised into a 30-item measure with a 12-item treatment stigma subscale.

**Conclusions:**

There is preliminary evidence demonstrating the reliability, validity and acceptability of the BACE. It can be used to ascertain key barriers to access to mental health care which may help to identify potential interventions to increase care seeking and service use. Further research is needed to establish its factor analytic structure and population norms.

## Background

### Avoidance of mental health care

A large proportion of people with mental illness do not receive care [1]. In Europe 27% of people experience mental illness (all disorders) each year, but 74% receive no treatment [2]. For mood, anxiety and substance use disorders severe enough to significantly interfere with everyday life, only 48% received any formal healthcare [3]. The figures for individuals with the same disorders in the USA are 31% with the mental disorders and 67% not treated [4]. In these countries much of the treatment gap can be accounted for by people not seeking or delaying seeking mental health care. In low and middle income countries there are even greater levels of undertreatment [5] due to both avoidance of health care and lack of care provision. Not seeking health care, or delayed care-seeking, may result in there being a longer period of untreated illness and, for psychotic illness, this is associated with worse outcomes such as having more symptoms, poorer functioning and quality of life and reduced likelihood of remission [6]. It may also contribute to adverse pathways to care such as the involvement of the criminal justice system and involuntary admissions under mental health legislation [7]. When individuals do seek or receive care they may subsequently drop out of services or may have low levels of engagement with services such as missing appointments or not seeking care in a crisis [8]. Therefore health care-seeking can be conceptualised as a process not restricted to the period before first contact with services. Whilst mental illness sometimes remits without professional care, avoidance of health care at any stage in this process may result in an individual not receiving effective treatments, which may contribute to the continuation of illness and the negative impacts that illness can bring, although this is not always so, particularly for less severe conditions.

The reasons why people with mental ill health sometimes avoid or delay seeking help from health services are numerous and include instrumental barriers such as not knowing where to go for help, or financial barriers, or they may be attitudinal such as perceived lack of effectiveness of treatments offered, thinking the problem will resolve itself, preferring to solve the problem on one’s own, and fear of being hospitalised against one’s will [9,10]. One reason that has received increasing research attention is the potential for stigma and discrimination to act as barriers to access to health care-seeking. Four reviews have examined the impact of stigma on access to mental health care and each concluded that it had a significant detrimental effect [11-14]. Prospective studies provide evidence that stigma may have a negative impact on service use [15,16]. Thus it is clear that both non-stigma-related (instrumental / attitudinal) and stigma-related barriers limit access to mental health care, but what is not currently known is the relative influence of these different types of barriers.

### Measuring mental health care-seeking

In the context of an ongoing systematic review on the association between stigma and health care we identified the following types of measure that have been used to assess mental health care seeking: previous / current actual service use; intention to seek health care; attitudes to health care seeking; recommendations regarding health care seeking for a vignette character; and barriers-based measures in which respondents are presented with a list of potential barriers and are asked whether, or not or to what extent, each has been a barrier for them. The advantage of barriers-based measures is that they indicate what prevents or delays individuals from health care-seeking and so may inform interventions to increase health care seeking and service use. We identified 23 studies that had assessed barriers to mental health care seeking [9,17-38].

A limitation of the existing barriers-based measures was that they did not always provide a comprehensive list of barriers. This was particularly true for stigma where measures sometimes simply referred to stigma as a barrier rather than enquiring about particular components of stigma (e.g. embarrassment / shame, concern about others’ disapproval, disclosure concerns, desire to avoid a stigma stereotype, and anticipated discrimination). Additionally the measures often had dichotomous response categories, when barriers are often experienced to a lesser or greater extent. Existing measures rarely encompassed service avoidance post-first contact. A final limitation is that few of the existing measures produced composite scores even though such scoring may be conceptually meaningful for some types of barrier e.g. stigma-related barriers.

### Measuring ‘treatment stigma’

‘Treatment stigma’ refers to the stigma and discrimination that individuals believe to be associated with receiving care for a mental health problem. We identified nine treatment stigma scales or subscales [27,39-46] from our on-going systematic review. The measures were in the main Likert attitude scales (measures with statements followed by ‘strongly agree’, ‘agree’ etc.). Only three measures were barriers-based [40,43,45]. A strength of a barriers-based measure of treatment stigma is that it is more tied to behaviour than attitudinal measures and permits the influence of stigma-related barriers to be compared to that of other types of barriers. However the existing barriers-based measures of treatment stigma cannot readily be used with general mental health populations as one is for adolescents [40], one has been used predominantly in military settings [43] and one mainly in the context of psychotherapy [45]. Furthermore there is no existing measure of treatment stigma which is applicable to all mental health conditions, all types of mental health care and all types of stigma. For example, some measures focus solely on specific conditions such as depression [27,44,46]; some refer solely to psychological help [41-43,45] and some to care from a psychiatrist [46]. Some measures exclusively assess the internalised stigma associated with receiving mental health care [42,44]; one focuses on the public stigma associated with psychological care for mental ill health [41], and one measures anticipated discrimination [46].

### Need for a new measure

We conclude from the overview of existing research and issues discussed above that there is a need for a comprehensive measure of barriers to access to professional care for mental ill health that has nominal rather than categorical response categories. In addition there is a currently unmet need for a barriers-based measure of treatment stigma that may be completed by individuals with any type of mental health problem, regarding any type of professional care, and covering all types of treatment stigma. We believe that a measure that encompasses health-care-seeking post- and well as pre- first receipt of care is needed to better reflect the fact that care-seeking is a process. The measure described here meets these three sets of requirements within one measure.

### Aims

This study aimed to:

1) develop a comprehensive self-complete measure - the Barriers to Access to Care Evaluation (BACE) - for assessing barriers to care-seeking for mental ill health, encompassing care avoidance post- as well as pre-contact with services;

2) develop from a subset of the BACE items a subscale for assessing the stigma associated with receiving mental health care (treatment stigma);

3) establish the psychometric properties of the BACE and its treatment stigma subscale;

4) present preliminary evidence about the prevalence of stigma-related and non-stigma-related barriers to care-seeking for mental ill health reported by a sample of adults recently or currently using secondary mental health services.

## Methods

### Design

The design is a four-stage psychometric validation study. Stage one involved the collation and reduction of barriers items from the existing literature and the creation of a draft measure. In stage two an expert panel revised the measure. Stage three was a cross-sectional survey repeated for test-retest reliability. This was embedded within a survey whose primary purpose was to develop another scale, the Questionnaire on Anticipated Discrimination (QUAD). Stage four was the finalisation of the measure based on psychometric findings from the survey.

### Sample and recruitment

As the BACE is intended for completion by individuals at any stage in the health care seeking process including those who have not and who have already received mental health care, we made a pragmatic decision to use the latter type of sample, taking the opportunity to include the preliminary BACE scale in the QUAD study. Inclusion criteria for the QUAD study, and consequently also the present study, were: 1) Having received care from secondary mental health services in the 12 months or currently; 2) aged 18 or over; and 3) access to the internet as the survey was to be completed online. Participants were recruited through the following routes: emails to members who participated in a previous phase of the QUAD development study in our research programme (all living in South-East England); and advertisements on websites and social networks (Facebook, Twitter) of England’s national anti-discrimination programme (Time to Change) and of two national UK mental health charities (Rethink, Mental Health Foundation). The target sample size was 100 (with 50 completing the survey a second time for assess test-retest reliability) and was based on the numbers required to perform the analyses needed for the psychometric validation of the QUAD.

### Ethics

The QUAD study and an amendment permitting the inclusion of the BACE v2, and the Stigma Scale for Seeking Psychological Help [41] were approved by the King’s College London Psychiatry, Nursing and Midwifery Research Ethics Sub-Committee (ref: PNM/09/10-103).

### Procedure

The BACE scale was developed through an iterative process. In stage one, one author (SC) collated barriers items from the 23 barriers studies [9,17-38] that we identified in our on-going systematic review on the association between stigma and health care seeking. These studies included samples of individuals with a variety of mental health problems and sociodemographic characteristics, who had and had not previously accessed care for mental health problems, as well as some general population studies. This initial method of sourcing items was chosen to capitalise on the large body of existing work in this area and to avoid duplication of effort. Items that were identical to other items, not relevant to a general population, ambiguous or rarely endorsed in the studies reporting them were then excluded from the item pool. When items used different words to refer to the same barrier, one item was selected, or the items were rephrased into a single new item. The research team then added items not yet covered that were known to be important in the qualitative and theoretical research literature on health care seeking. This set constituted the items for the BACE v1. The research team devised a root question: ‘Have any of these issues ever stopped, delayed or discouraged you from getting, or continuing with, professional care for a mental health problem?’. Professional care was defined as ‘care from such staff as a GP (family doctor), community mental health team (e.g. care coordinator, mental health nurse or mental health social worker), psychiatrist, counsellor, psychologist or psychotherapist’. The response categories were ‘This has stopped, delayed or discouraged me not at all / a little / quite a lot / a lot’.

In stage two BACE v1 was sent to an expert panel for comment. The panel comprised three people with experience of mental illness (various diagnoses, all with experience of both using and avoiding mental health care), three social scientists working in the field of stigma and / or help-seeking, three clinicians (two psychiatrists and a mental health nurse) and a lay person with no post-16 education to check for clarity and ease of understanding. The feedback was used to delete, add and rephrase items to create the BACE v2. Each item was designated by the research team, on a conceptual basis, as being either a stigma-related or non-stigma-related barrier to care seeking.

In stage three participants completed the BACE v2 and other measures in an anonymised online survey. Participants were invited to recomplete the BACE v2 two weeks later, also online. The fourth stage of scale development was to modify the BACE v2 on the basis of the psychometric findings and further discussion within the research team into the BACE v3.

### Measures

Three measures relevant to this study were included in the survey: (i) the Barriers to Access to Care Evaluation (BACE v2); (ii) the Stigma Scale for Receiving Psychological Help (SSRPH) [41]; and (iii) the Internalised Stigma of Mental Illness (ISMI) [47]. The latter two were used for assessing the convergent validity of the BACE. The Barriers to Access to Care Evaluation (BACE v2) is a 36-item measure scored from 0 (not at all) to 3 (a lot) with higher scores indicating a greater barrier. The BACE treatment stigma subscale score is the mean of stigma-related barriers ratings. It ends with space for free text entries describing two other barriers participants may have experienced. The Stigma Scale for Receiving Psychological Help (SSRPH) [41] is a 5-item measure of treatment stigma focusing predominantly on social stigma. Golberstein’s [48] adapted version referring to any type of mental health treatment was used. It has four response categories from strongly disagree to strongly agree, with a high scores indicating greater treatment stigma. Its internal consistency is alpha = 0.72 [41]**.** The Internalised Stigma of Mental Illness (ISMI) scale is a 29-item measure that assesses mental health service users’ experience of internalised stigma [47]. It has four response categories from strongly disagree to strongly agree and high scores indicate high internalised stigma. It has strong internal consistency (α = 0.90) and test–retest reliability (r = 0.92) [47]. In addition participants were asked ‘Please choose one number between 1 (very poor) and 10 (very good) to show your overall opinion of the BACE questionnaire?’ and to add free text comments about the measure. There were further items on sociodemographic and illness characteristics.

### Analysis

Data were analysed using SPSS v15 [49] and Stata v10.1 [50]. There were no missing data as the online survey was set to preclude this.

#### Reliability

To assess the test-retest reliability of individual items weighted intra-class correlation co-efficients were calculated with kappa’s above 0.4 indicating moderate agreement [51] as the criterion for acceptable reliability. Lin’s concordance statistic (ρ_c_) was used to calculate the overall test-retest reliability for the treatment stigma subscale [52], with a criterion of Lin’s ρ_c_>0.70 used to indicate acceptable reliability. The internal consistency of the treatment stigma subscale was assessed using Cronbach’s alpha, with a value above 0.7 but not higher than 0.9 taken to indicate good internal consistency [53].

#### Validity

Content validity is the extent to which a measure comprehensively covers domains of interest [54] and was assured by the development of the measure having incorporated the extant literature, and the perspectives of people with mental illness and professional experts. A further indication of this type of validity was provided by content analysis of free text answers about other barriers. Construct validity was assessed by examining convergent validity [55], hypothesising that the treatment stigma subscale would be significantly positively correlated with both the SSRPH and the ISMI.

#### Acceptability

Two aspects of the acceptability of the measure were considered: respondent opinion and readability. Descriptive statistics on respondents’ overall ratings of the BACE.v2 were calculated. The readability of the BACE v3 was assessed using the Flesch Reading Ease score and Flesch-Kincaid Grade level functions within Microsoft Word which assess readability based on the syllabic and sentence structure of the text [56]. Scores for the former range from 0–100 with higher scores meaning easier to read and scores of 60-70 representing acceptable scores for documents for general adult populations. The latter provides the US educational grade to which the material is most appropriate [56].

#### Finalising the BACE v2 into the BACE v3

Items which were endorsed as a major barrier by less than 10% of the sample were considered for removal as were items that were very highly correlated with other items (Spearman’s rho > 0.7) and those with poor or slight test-retest agreement (Kappa ≤ 0.2 [57]. Conceptual and wording issues were also further considered at this stage and amendments made.

#### Prevalence of stigma-related and non-stigma-related barriers

Percentages endorsing each item as a barrier (to any degree) or as a major barrier (rated as ‘a lot’) were calculated together with mean ratings. Barriers were then ranked by percentage rating them as a major barrier to show the relative prevalence of stigma-related and non-stigma-related barriers.

## Results

### Participants

117 individuals completed the survey, and 59 of these participants recompleted the BACE to provide test-retest reliability data. Those providing the test-retest data did not differ significantly from the remainder of the sample in age, gender, ethnicity, education, employment status, age at first treatment, and hospital admittance for psychiatric treatment, although those with non-psychotic conditions were more likely to be in the retest sample (66% vs 34%, ^2^ = 5.062, p = 0.024) compared to those with psychotic conditions. The characteristics of the sample are shown in Table 1. Participants had a mean age of 36 (range 18 to 70), 80% were female, the majority (87%) reported their ethnicity as White British, and 42% were in full- or part-time employment. The most common self-reported primary diagnoses were depression (34%) and bipolar disorder (31%). Forty six percent had been hospitalised for a mental health problem.

**Table 1:** Participant sociodemographic and clinical characteristics

		**N**	**%**
Gender (n = 117)	Male	24	20.5
	Female	93	79.5
Ethnicity (n = 117)	White British	102	87.2
	White Irish	5	4.3
	Other white background	5	4.3
	Black British / Black African	2	1.8
	Indian / Bangladeshi	2	1.8
	White and Asian	1	0.9
Age (n = 115)	Mean (sd) = 36.1(11.1)		
Highest level of education (n = 117)	Higher education qualification / degree	61	54.7
	Vocational qualification	16	13.7
	A levels	17	14.5
	GCSE / O level / CSE	19	16.2
	No formal qualifications	1	0.9
Employment status (n = 117)	Work full-time	33	28.2
	Work part-time	16	13.7
	Volunteer	19	16.2
	Looking after own children	2	1.7
	Student	11	9.4
	Retired	1	0.9
	Not working	35	30.0
Relationship status (n = 116)	Single	52	44.8
	Married / civil partnership / cohabiting	46	39.7
	Divorced or separated	16	13.8
	Widowed	2	1.7
Any children (including adult and non-resident children) (n = 116)	Yes	34	29.3
	No	82	70.7
Self-reported diagnosis (if more than one, first listed) n = 107)	Schizophrenia / schizoaffective disorder	5	4.7
	Bipolar disorder	33	30.8
	Depression	36	33.6
	Anxiety disorder	13	12.1
	Personality disorder	17	15.9
	Other	3	2.8
Ever admitted to hospital for psychiatric treatment? (n = 116)	Yes	53	45.7
	No	63	54.3
Any involuntary hospital admissions? (n = 114)	Yes	12	10.5
	No	102	87.2
Years since first treatment for mental health problem (n = 104)	Mean(sd) = 12.9 (9.4)		

### Development of the BACE v1 and v2

There were 172 barriers items in the 23 papers assessing barriers. This was reduced to 30 items following deletion and amalgamation by the research team. The team identified eight additional items from their knowledge of the research literature. Consequently the BACE v1, sent to the expert panel, had 38 items. Four of these items were deleted following feedback from the panel because they were viewed as ambiguous or already covered by a similar item. Two new items (20 and 21 in Table 2) were added at the expert panel’s suggestion making the BACE v2 a 36 item measure. Minor rewording was made in five items (revised items 3, 12, 16, 24 and 34 in Table 2), and two items were reworded for conceptual reasons (revised items 9 and 11 in Table 2). The research team designated 13 of the BACE v2 items as stigma-related, 23 as non-stigma related barriers, as shown in Table 2. One item (‘Difficulty taking time off work’) was viewed as potentially both instrumental and stigma-related because it risked disclosing the mental illness. A further two items (‘Concerns about the confidentiality of the information I share’ and ‘Dislike of talking about my feelings, emotions or thoughts’) were viewed as potentially both attitudinal and stigma-related. A decision was made to only designate items stigma-related if when there was no alternative potential designation for the barrier. The items that were designated stigma-related spanned anticipated discrimination in employment (items 6 and 33 in Table 2) and in relation to parenting (item 29), social stigma (items 9, 16 and 31), disclosure concerns (items 19 and 25), stereotypes (items 3, 14 and 21), internalised stigma (item 11) and stigma by association (item 26).

**Table 2:** **Mean scores, frequencies and ranks for each barrier in the BACE v2 (n = 117**^**a**^**)**

Item no.	Barrier	Barrier type^b^	Mean (sd)	%reporting barrier to any degree	%reporting as major barrier (‘a lot’)	Rank (1 = item has highest proportion rating as a major barrier)
1.	Being unsure where to go to get professional care	N-S	1.47 (1.05)	77.8	19.7	21
2.	Wanting to solve the problem on my own	N-S	1.84 (1.02)	88.0	32.5	6
3.	Concern that I might be seen as weak for having a mental health problem	S	1.75 (0.99)	86.3	25.6	12
4.	Difficulty taking time off work	N-S	1.75 (1.14)	78.6	34.2	3
5.	Fear of being put in hospital against my will	N-S	1.44 (1.08)	75.2	20.5	19
6.	Concern that it might harm my chances when applying for jobs	S	1.98 (1.01)	89.7	39.3	1
7.	Problems with transport or travelling to appointments	N-S	1.24 (1.13)	65.0	18.8	23
8.	Thinking the problem would get better by itself	N-S	1.51 (1.08)	76.9	22.2	15
9.	Concern about what my family might think or say	S	1.37 (1.10)	71.8	20.5	20
10.	Being unhappy with the available services	N-S	1.72 (1.05)	84.6	29.1	8
11.	Feeing embarrassed or ashamed	S	1.46 (1.00)	82.9	19.7	22
12.	Preferring to get alternative forms of care (e.g. spiritual care, non-Western healing / medicine, complementary therapies)	N-S	0.49 (0.81)	33.3	4.3	35
13.	Not being able to afford the financial costs involved	N-S	1.30 (1.18)	63.2	22.2	16
14.	Concern that I might be seen as ‘crazy’	S	1.38 (1.02)	78.6	16.2	25
15.	Thinking that professional care probably would not help	N-S	1.44 (1.05)	35.0	21.4	17
16.	Concern that I might be seen as a bad parent (data from parent subsample, n = 34)	S	1.94 (1.04)	88.2	38.2	2
17.	Professionals from my own ethnic or cultural group not being available	N-S	0.36 (0.81)	19.7	5.1	34
18.	Being too unwell to ask for help	N-S	1.72 (1.11)	82.9	33.3	4
19.	Concern that people I know might find out	S	1.29 (1.01)	73.5	13.7	27
20.	Dislike of talking about my feelings, emotions or thoughts	N-S	1.50 (1.10)	78.6	26.5	11
21.	Concern that people might not take me seriously if they found out I was having professional care	S	1.37 (0.98)	78.6	14.5	26
22.	Having no one who could come to appointments with me	N-S	0.90 (1.00)	53.8	9.4	31
23.	Lack of trust in professionals who provide professional care for mental health problems	N-S	1.55 (1.13)	78.6	29.1	9
24.	Concerns about the treatments available (e.g. medication side effects)	N-S	1.68 (1.00)	85.5	23.9	13
25.	Not wanting a mental health problem to be on my medical records	S	1.54 (1.19)	72.6	30.8	7
26.	Concern that it might bring shame or disapproval on my family	S	0.89 (1.06)	49.6	11.1	30
27.	Having had previous bad experiences with professional care for mental health	N-S	1.56 (1.21)	72.6	33.3	5
28.	Preferring to get help from family or friends	N-S	0.58 (0.80)	41.1	2.6	36
29.	Concern that my children may be taken into care or that I may lose access or custody (parent subsample, n = 34)	S	1.41 (1.18)	67.6	23.5	14
30.	Thinking I did not have a problem	N-S	1.15 (1.07)	66.7	17.1	24
31.	Concern about what my friends might think or say	S	0.92 (0.95)	58.1	6.8	33
32.	Thinking appointments take too much time or are inconvenient	N-S	0.81 (0.97)	50.4	8.5	32
33.	Concern that it might harm my career or chances of promotion	S	168 (1.06)	82.1	27.4	10
34.	Having problems with childcare while I receive professional care (parent subsample, n = 34)	N-S	0.90 (1.11)	47.1	11.6	29
35.	Having no one who could help me get professional care	N-S	1.03 (1.04)	60.7	12.8	28
36.	Concerns about the confidentiality of the information I share	N-S	1.24 (1.18)	61.5	21.4	18

a. Except where otherwise stated.

b. stigma-related (S); non-stigma-related (N-S).

### Distribution of the BACE treatment stigma subscale

BACE treatment stigma scores varied from 0 to 3 with a mean of 1.43 (sd 0.73). The BACE treatment stigma scale was normally distributed (Kolmogrov-Smirnov Z = 0.86, p = 0.454), therefore parametric statistics are appropriate for use with this scale.

### Reliability

The majority (22/36) of the BACE v2 items had weighted kappa values from 0.61 to 0.80 indicating substantial agreement between test and retest [51]; two items (12 and 29 of the BACE v2 shown in Table 2) had values above 0.8 indicating almost perfect agreement; 11 items had values between 0.41 and 0.60 indicating moderate agreement, and one (item 3, ‘Concern about being seen as weak for having a mental health problem’) had a kappa of 0.346 meaning fair reliability, but not reaching the pre-specified criterion of 0.4. Lin’s concordance statistic for the treatment stigma subscale was ρ_c_ = 0.816 exceeding the criterion of 0.70 for acceptable test-retest reliability. The Cronbach’s alpha value for the treatment stigma subscale was 0.89 indicating good internal consistency.

### Validity

Twenty two participants gave free text answers in response to questions about other barriers. Content analysis the 36 additional barriers revealed 20 were already covered by the existing items, and 10 were proposed by one respondent only, thereby indicating good content validity. Provider delay and providers not responding to requests for help were mentioned as a barrier by four people, which suggests this is an aspect for further study. The hypothesised significant positive correlation between the BACE treatment stigma subscale and the SSRPH [41] was supported (r = 0.30, p = 0.001), as was the same hypothesised relationship between the BACE treatment stigma subscale and the Internalised Stigma of Mental Illness Scale [47] (r = 0.40, p < 0.001). Thus the subscale has convergent and hence construct validity.

### Acceptability

Respondents gave a median overall evaluation rating of the BACE of 8 (IQR 7-9) on the 10-point scale, indicating a positive respondent opinion of the measure. The Flesch Reading Ease score for the BACE v3 was 78.8, indicating it is easier to read than documents at the general population level of 60-70. Its Flesch-Kincaid Grade Level was 5.9 indicating that it can be understood by the average 11 to 12 year-old.

### Finalisation of the BACE v3

The research team deleted five items and amalgamated two items on the basis of item analysis and conceptual discussion, as well as making some final wording changes. Items 10 and 23 were removed because they were very highly correlated with another variable (rho > 0.7) and were highly correlated (rho > 0.5) with a further three variables. No other variables had this degree of inter-correlation. Items 22 and 32 were deleted because fewer than 10% of respondents rated the issue as a major barrier. Three other items (12, 17, 28, and 31) also met this criterion but were retained, the first two because they were relatively highly endorsed by non-White participants; the third because the literature suggests that informal care seeking is a common factor in delay in seeking professional care [57] and perhaps would be more evident in less unwell populations; and the fourth because concern about stigma from friends has been shown to be an important factor in young populations [22]. Item 3 which did not meet the moderate test-retest reliability criterion was considered for removal but retained as its reliability level was ‘fair’ [51] and because it was a relatively highly rated barrier (ranked 12/36). Two stigma-related items (9 and 26) were amalgamated with each other because only 11% rated the latter as a major barrier and because it had some conceptual similarity to the former. Item 36 was removed because it was considered to have a large conceptual overlap with item 25. Thus the BACE v3 has 30 items including 12 that are stigma-related. Following team discussion conceptual rewording was made to items 9, 29, 31 and 33 and minor rewording made to item 10. When asked for free text comments on the BACE, several respondents pointed out the need to have a ‘not applicable box’. These have now been added to the items referring to employment and to children. The scoring for the treatment stigma subscale was consequently amended from the mean rating to the mean of ratings for applicable items. This scoring method has been used successfully for other measures with varying numbers of applicable life domains, such as the DISC [58]. This finalised version of the BACE can be seen in Additional file 1.

### Prevalence of stigma-related and non-stigma-related barriers to access to care

The prevalence of stigma-related and non-stigma-related barriers to care-seeking for mental ill health reported by this sample is shown in Table 2. The top two barriers – ‘concern that it might harm my chances when applying for jobs’ (item 6) and ‘concern that I might be seen as a bad parent’ (item 16) - were both stigma-related with 39% and 38% reporting these as major barriers respectively and 89% and 88% experiencing them to some degree. The next most highly ranked barriers were difficulty taking time off work, being too unwell to ask for help, having had previous bad experiences, wanting to solve the problem on one’s own, and not wanting a mental health problem on one’s medical records with only the latter being a designated stigma-related barrier. Some types of stigma-related barriers were relatively rarely endorsed such as concern about it bringing shame or disapproval on one’s family and about what friends might think or say with 11% and 7% reporting these as a major barrier respectively.

## Discussion

### Strengths and limitations

The main strengths of the study are the iterative detailed process of measure development and its relatively broad assessment of psychometric properties. The key strengths of the new measure include the comprehensiveness of the list of potential barriers; its format making it possible to use in all care settings and with all mental health conditions; that it encompasses care avoidance post- as well as pre-contact with services; and its incorporation of a barriers-based treatment stigma scale, encompassing more types of stigma than existing measures. A benefit of the measure is that it gives the flexibility to assess the extent of a barrier (mean score), and the frequency with which a barrier is experienced to any degree, and as a major barrier. The study is limited by its self-selected sample recruited through specific organisations, and one of the recruitment pathways was the website of an anti-discrimination programme (Time to Change) which may have increased the ratings of stigma-related barriers. The sample was also restricted to those with internet access. Men, Black and minority ethnic groups, and those with schizophrenia / schizoaffective disorder diagnoses were under-represented in the sample, although with only 42% in paid employment the sample is socioeconomically diverse. The psychometric and prevalence data may have been different had we used a sample who had never accessed care. Because the survey software was set up so as to require responses to all items, we have no information about the extent or patterns of missing data that would apply with the BACE. Such data would have provided useful additional information about the acceptability of the BACE and the content validity of its items. Our strategy of selecting items from existing barriers measures identified through a systematic review on stigma and healthcare seeking may have missed some non-stigma-related barriers, although the final measure contains 18 such items and so appears comprehensive. A further drawback is that, in the present study, we were not able to undertake a full psychometric assessment, which would include, for example, a factor analysis and examination of responsiveness. It is a limitation that the initial selection of items was performed by one researcher rather two*.* Lastly it is possible that a person who has more applicable life domains will have a greater opportunity to experience stigma and this could affect their treatment stigma score, although the use of the mean-of-applicable-items scoring method is likely to minimise any such effects.

### Applications

The BACE may be used to identify key barriers to care experienced by people who currently use, or have recently used, secondary mental health services, and it has potential utility for use with general population samples. This may help to identify potential interventions to increase care seeking and service use and reduce the duration of untreated illness. It might also be used to assess change in barriers to care after the introduction of such interventions. The 12-item treatment stigma subscale may be used on its own, or as part of the 30-item BACE scale depending on the requirements of the particular research questions being addressed. The BACE v3 is the version recommended for use and is available, together with the BACE manual, from the authors.

### Implications for future research

Further data is needed on the performance of the BACE v3 in a more representative sample, and one that is large enough for factor analysis. Our group is currently undertaking such work in the MIRIAD study (part of the SAPPHIRE Research Programme on Stigma and Discrimination in Mental Health, http://www.sapphire.iop.kcl.ac.uk). This study will also inform us about the psychometrics of the two additional items suggested by the participants of the present study (‘Having asked for help but not receiving it’ and ‘Having asked for help but having to wait a long time before receiving it’) and about two ethnicity-related barriers added because of that study’s particular focus (‘Concern that mental health staff will not understand cultural issues that are important to me’ and ‘Concern that I will be treated unfairly by mental health staff or services because of my ethnic background’). With severe mental illness, family members often play a major role in health care-seeking on behalf of the person affected [59]. In the MIRIAD study we are using an adapted version of the BACE scale with informal caregivers and will report psychometric findings on the reliability and validity of this adaptation when data collection is complete. Further psychometric analysis is warranted to establish the responsiveness of the BACE and its treatment stigma subscale; and to explore the factor structure of the latter. There is a need for evidence about the scale’s performance in a community sample. As the participants in our study had accessed mental health care the level of barriers they reported is likely to be lower than that of those with mental health needs who have not accessed care, and the pattern of this latter group may be different. Further research is needed to elucidate levels and patterns of barriers in such populations. Lastly, the BACE is currently being used in the UK, with planned usage in Switzerland and Nepal, however, further evidence about its suitability for other international contexts would be welcome.

## Conclusions

We have developed a new measure of barriers to access to care for mental ill health - the BACE – and have demonstrated preliminary evidence of its reliability, validity and acceptability. It was developed by systematically reducing 172 barrier items identified the extant literature to 38 (v1), then 36 (v2), then 30 (v3) to create a comprehensive self-complete measure assessing barriers to access to mental health care. Through this process we identified a broad spectrum of stigma-related barriers encompassing anticipated discrimination, social stigma, disclosure concerns, stereotypes, internalised stigma and stigma by association. The new measure incorporates a treatment stigma subscale for assessing the stigma associated with receiving mental health care. The BACE scale and its treatment stigma subscale were found to have good test-retest reliability, internal consistency, and content and construct validity. The measure was positively evaluated by participants and had readability levels appropriate for populations with mental illness. The study also provided preliminary evidence about the prevalence of stigma-related and non-stigma-related barriers to care-seeking for mental ill health reported by a sample of adults recently or currently using secondary mental health services. The most frequently endorsed barriers were of both types, although the top two were stigma-related. This attests to the importance of stigma as a key factor limiting access to mental health care [11-14,60].

## Competing interests

The authors declare that they have no competing interests.

## Authors’ contributions

SC designed and led the study, drafted the versions of the questionnaire, conducted the main analysis and drafted the manuscript. EB contributed to study design, questionnaire development, and data interpretation, and collected the data and performed the statistical analysis of the test-retest data test-retest analyses. DJ contributed to the literature review and data interpretation. CH, SLH and GT contributed to study design, questionnaire development, and data interpretation. All authors critically revised the draft manuscript, and read and approved the final manuscript.

## Pre-publication history

The pre-publication history for this paper can be accessed here:

http://www.biomedcentral.com/1471-244X/12/36/prepub

## Supplementary Material

Additional file 1 Barriers to Access to Care Evaluation (BACE v3). Click here for file
