# Supplementary material for: Development and psychometric properties the Barriers to Access to Care Evaluation scale (BACE) related to people with mental ill health
Source: BMC Psychiatry. 2012 Jun 20;12:36. doi: 10.1186/1471-244X-12-36 (PMC3379935; doi:10.1186/1471-244X-12-36)
Supplement: Additional file 1 — Barriers to Access to Care Evaluation (BACE v3). [file 1471-244X-12-36-S1.doc]

**Barriers to Access to Care Evaluation (BACE v3)^1^**

Below you can see a list of things which can stop, delay or discourage people from getting professional care for a mental health problem, or continuing to get help. By professional care we mean care from such staff as a GP (family doctor), community mental health team (e.g. care coordinator, mental health nurse or mental health social worker), psychiatrist, counsellor, psychologist or psychotherapist.

**Have any of these issues ever stopped, delayed or discouraged you from getting, or continuing with, professional care for a mental health problem?**

***Please circle one number on each row to indicate the answer that best suits you****.*

For ‘not applicable’ e.g. if it is a question about children and you do not have children, please cross the Not applicable box.

|  | **Issue** | This has stopped, delayed or discouraged me  **NOT AT ALL** | This has stopped, delayed or discouraged me  **A LITTLE** | This has stopped, delayed or discouraged me  **QUITE A LOT** | This has stopped, delayed or discouraged me  **A LOT** |
| --- | --- | --- | --- | --- | --- |
| 1. | Being unsure where to go to get professional care | 0 | 1 | 2 | 3 |
| 2. | Wanting to solve the problem on my own | 0 | 1 | 2 | 3 |
| 3. | Concern that I might be seen as weak for having a mental health problem | 0 | 1 | 2 | 3 |
| 4. | Fear of being put in hospital against my will | 0 | 1 | 2 | 3 |
| 5. | Concern that it might harm my chances when applying for jobs  Not applicable **□** | 0 | 1 | 2 | 3 |
| 6. | Problems with transport or travelling to appointments | 0 | 1 | 2 | 3 |
| 7. | Thinking the problem would get better by itself | 0 | 1 | 2 | 3 |
| 8. | Concern about what my family might think, say, do or feel | 0 | 1 | 2 | 3 |
| 9. | Feeing embarrassed or ashamed | 0 | 1 | 2 | 3 |
| 10. | Preferring to get alternative forms of care (e.g. traditional / religious healing or alternative / complementary therapies) | 0 | 1 | 2 | 3 |
| 11. | Not being able to afford the financial costs involved | 0 | 1 | 2 | 3 |
| 12. | Concern that I might be seen as ‘crazy’ | 0 | 1 | 2 | 3 |
| 13. | Thinking that professional care probably would not help | 0 | 1 | 2 | 3 |
| 14. | Concern that I might be seen as a bad parent  Not applicable **□** | 0 | 1 | 2 | 3 |
| 15. | Professionals from my own ethnic or cultural group not being available | 0 | 1 | 2 | 3 |
| 16. | Being too unwell to ask for help | 0 | 1 | 2 | 3 |
| 17. | Concern that people I know might find out | 0 | 1 | 2 | 3 |
| 18. | Dislike of talking about my feelings, emotions or thoughts | 0 | 1 | 2 | 3 |
| 19. | Concern that people might not take me seriously if they found out I was having professional care | 0 | 1 | 2 | 3 |
| 20. | Concerns about the treatments available (e.g. medication side effects) | 0 | 1 | 2 | 3 |
| 21 | Not wanting a mental health problem to be on my medical records | 0 | 1 | 2 | 3 |
| 22. | Having had previous bad experiences with professional care for mental health | 0 | 1 | 2 | 3 |
| 23. | Preferring to get help from family or friends | 0 | 1 | 2 | 3 |
| 24. | Concern that my children may be taken into care or that I may lose access or custody without my agreement  Not applicable **□** | 0 | 1 | 2 | 3 |
| 25. | Thinking I did not have a problem | 0 | 1 | 2 | 3 |
| 26. | Concern about what my friends might think, say or do | 0 | 1 | 2 | 3 |
| 27. | Difficulty taking time off work  Not applicable **□** | 0 | 1 | 2 | 3 |
| 28. | Concern about what people at work might think, say or do  Not applicable **□** | 0 | 1 | 2 | 3 |
| 29. | Having problems with childcare while I receive professional care  Not applicable **□** | 0 | 1 | 2 | 3 |
| 30. | Having no one who could help me get professional care | 0 | 1 | 2 | 3 |

1. Barriers to Care Evaluation (BACE) Scale (v3) Institute of Psychiatry, King’s College London © 2011. For permission to use and a copy of the manual, please contact Dr Sarah Clement [sarah.clement@kcl.ac.uk](mailto:sarah.clement@kcl.ac.uk) or Professor Graham Thornicroft, [graham.thornicroft@kcl.ac.uk](mailto:graham.thornicroft@kcl.ac.uk).
